# Cellular and Molecular Pathophysiology of Gestational Diabetes

**DOI:** 10.3390/ijms252111641

**Published:** 2024-10-30

**Authors:** Johnatan Torres-Torres, Irma Eloisa Monroy-Muñoz, Javier Perez-Duran, Juan Mario Solis-Paredes, Zaira Alexi Camacho-Martinez, Deyanira Baca, Salvador Espino-y-Sosa, Raigam Martinez-Portilla, Lourdes Rojas-Zepeda, Hector Borboa-Olivares, Enrique Reyes-Muñoz

**Affiliations:** 1 Department of Reproductive and Perinatal Health Research, Instituto Nacional de Perinatología Isidro Espinosa de los Reyes, Mexico City 11000, Mexico; 2 Obstetric and Gynecology Department, Hospital General de México Dr. Eduardo Liceaga, Mexico City 06720, Mexico; 3 Centro de Investigacion en Ciencias de la Salud, Universidad Anahuac Mexico, Campus Norte, Huixquilucan 52786, Mexico; 4 Maternal-Fetal Department, Instituto Materno Infantil del Estado de Mexico, Toluca 50170, Mexico; 5 Community Interventions Research Branch, Instituto Nacional de Perinatología Isidro Espinosa de los Reyes, Mexico City 11000, Mexico; 6 Research Division, Instituto Nacional de Perinatología Isidro Espinosa de los Reyes, Mexico City 11000, Mexico

**Keywords:** gestational diabetes, insulin resistance, mitochondrial dysfunction, oxidative stress, chronic inflammation, placental dysfunction

## Abstract

Gestational diabetes (GD) is a metabolic disorder characterized by glucose intolerance during pregnancy, significantly impacting maternal and fetal health. Its global prevalence is approximately 14%, with risk factors including obesity, family history of diabetes, advanced maternal age, and ethnicity, which are linked to cellular and molecular disruptions in glucose regulation and insulin resistance. GD is associated with short- and long-term complications for both the mother and the newborn. For mothers, GD increases the risk of developing type 2 diabetes, cardiovascular diseases, and metabolic syndrome. In the offspring, exposure to GD in utero predisposes them to obesity, glucose intolerance, and metabolic disorders later in life. This review aims to elucidate the complex cellular and molecular mechanisms underlying GD to inform the development of effective therapeutic strategies. A systematic review was conducted using medical subject headings (MeSH) terms related to GD’s cellular and molecular pathophysiology. Inclusion criteria encompassed original studies, systematic reviews, and meta-analyses focusing on GD’s impact on maternal and fetal health, adhering to PRISMA guidelines. Data extraction captured study characteristics, maternal and fetal outcomes, key findings, and conclusions. GD disrupts insulin signaling pathways, leading to impaired glucose uptake and insulin resistance. Mitochondrial dysfunction reduces ATP production and increases reactive oxygen species, exacerbating oxidative stress. Hormonal influences, chronic inflammation, and dysregulation of the mammalian target of rapamycin (mTOR) pathway further impair insulin signaling. Gut microbiota alterations, gene expression, and epigenetic modifications play significant roles in GD. Ferroptosis and placental dysfunction primarily contribute to intrauterine growth restriction. Conversely, fetal macrosomia arises from maternal hyperglycemia and subsequent fetal hyperinsulinemia, resulting in excessive fetal growth. The chronic inflammatory state and oxidative stress associated with GD exacerbate these complications, creating a hostile intrauterine environment. GD’s complex pathophysiology involves multiple disruptions in insulin signaling, mitochondrial function, inflammation, and oxidative stress. Effective management requires early detection, preventive strategies, and international collaboration to standardize care and improve outcomes for mothers and babies.

## 1. Introduction

Gestational diabetes (GD) is a metabolic complication that emerges during pregnancy, defined as diabetes diagnosed in the second or third trimester of pregnancy that was not clearly overt diabetes prior to gestation [1]. It is a condition that significantly affects both maternal and fetal health, leading to short- and long-term complications [2]. Globally, its prevalence is approximately 14% [3]. The development of GD is influenced by several risk factors, including obesity, family history of diabetes, advanced maternal age, and ethnicity, all of which play critical roles in the disease’s onset and progression [4,5]. Obesity is one of the most prominent risk factors for GD, as it is closely linked to insulin resistance and chronic inflammation. Excess adipose tissue in obese individuals triggers the release of pro-inflammatory cytokines, which disrupt insulin signaling and exacerbate glucose metabolism dysregulation [6,7]. A family history of diabetes suggests a genetic predisposition, with epigenetic modifications, such as DNA methylation and histone acetylation, further promoting insulin resistance and inflammation [8,9]. Advanced maternal age also contributes to GD as changes in trophoblast function, including mitochondrial dysfunction and increased reactive oxygen species (ROS). ROS are natural byproducts of normal cellular metabolism, and their levels are usually kept in check by cellular antioxidant systems; however, the excess of ROS impairs placental function and exacerbates insulin resistance [10,11]. Ethnicity also plays a crucial role in GD susceptibility. This disparity is often attributed to genetic and environmental factors that influence insulin sensitivity and glucose metabolism. In addition to genetic differences, cultural and lifestyle factors, such as diet and physical activity, can further modify the risk of developing GD across different ethnic groups [12,13].

Overall, GD results from a complex interplay of genetic, environmental, and physiological factors. This review aims to elucidate the complex cellular and molecular mechanisms of GD, which are key to developing effective therapies and improving maternal and fetal outcomes. It focuses on cellular signaling, metabolism, gut microbiota, gene expression, epigenetics, inflammation, oxidative stress, and the roles of placental and trophoblastic cells in GD.

## 2. Results

This review comprehensively explores the pathophysiology of GD from cellular and molecular perspectives. This detailed examination is structured to provide a step-by-step understanding of the underlying mechanisms that contribute to GD. Each section addresses specific aspects of the disorder, starting with disruptions in cellular signaling and metabolism and progressing through hormonal influences, inflammatory pathways, mitochondrial dysfunction, mammalian target of rapamycin (mTOR) pathway dysregulation, and the overexpression of the Klotho protein. Additionally, the roles of gut microbiota, gene expression, and epigenetic modifications in GD are elucidated, along with the impacts of ferroptosis and the critical functions of placental and trophoblastic cells (Table 1).

### 2.1. Insulin Signaling Pathway

**Figure 1:**
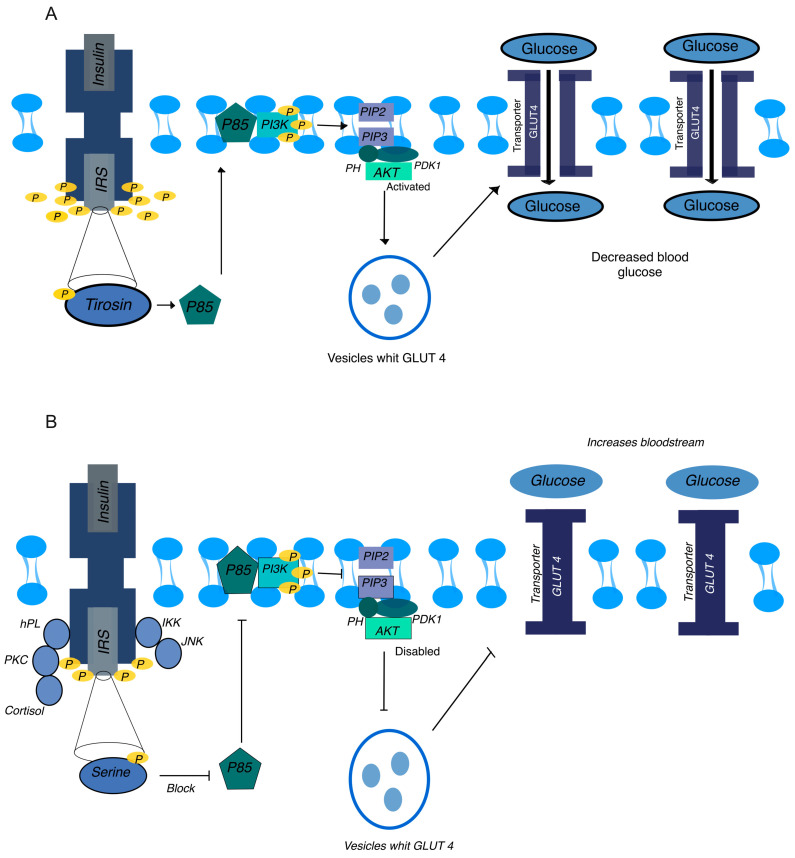
Insulin signaling pathway in normal and gestational diabetes (GD) conditions. (**A**) Normal insulin signaling. When insulin binds to its receptor, it triggers autophosphorylation on specific tyrosine residues, which creates docking sites for insulin receptor substrates (IRS-1/2). These substrates undergo tyrosine phosphorylation, activating the PI3K pathway. The active PI3K converts PIP2 into PIP3, which recruits and activates Akt at the cell membrane. Activated Akt then facilitates the translocation of GLUT4 vesicles to the cell membrane, allowing glucose uptake into the cell and reducing blood glucose levels. (**B**) Disrupted insulin signaling in GD. In gestational diabetes, elevated levels of hormones (e.g., human placental lactogen, cortisol) and pro-inflammatory cytokines (e.g., TNF-α, IL-6) induce aberrant serine phosphorylation of IRS-1/2 instead of tyrosine phosphorylation. This modification inhibits IRS function, leading to reduced activation of PI3K. Consequently, the conversion of PIP2 to PIP3 is impaired, decreasing Akt activation. The insufficient activation of Akt prevents GLUT4 vesicles from translocating to the cell membrane, thereby blocking glucose uptake. As a result, glucose remains in the bloodstream, contributing to insulin resistance and hyperglycemia typical of GD.

In GD, the normal insulin signaling pathway is disrupted, leading to impaired glucose uptake and insulin resistance. Insulin, a hormone essential for regulating blood glucose levels, binds to its receptor on the cell surface, triggering a cascade of phosphorylation events necessary for glucose regulation (Figure 1A).

Under normal conditions, insulin binding to its receptor on the cell surface triggers autophosphorylation of the receptor at specific tyrosine residues and phosphorylation of the insulin receptor substrates (IRS-1 and IRS-2) at tyrosine residues [14,15]. Phosphorylation of these substrates is essential for activation of the downstream phosphoinositide 3-kinase (PI3K) signaling pathway [14,16].

Phosphorylated IRS-1 and IRS-2 interact with the p85 regulatory subunit of PI3K, leading to the conversion of phosphatidylinositol-4,5-bisphosphate (PIP2) into phosphatidylinositol-3,4,5-triphosphate (PIP3). PIP3 acts as a lipid second messenger, recruiting proteins with pleckstrin homology (PH) domains to the cell membrane, including Akt, also known as protein kinase B [17]. Akt is subsequently phosphorylated and activated by phosphoinositide-dependent kinase-1 (PDK1), triggering signaling events crucial for glucose metabolism [18].

One of the critical actions of activated Akt is to facilitate the translocation of glucose transporter type 4 (GLUT4) vesicles from intracellular compartments to the cell membrane. GLUT4 is a protein that transports glucose into the cell, and its movement to the cell membrane is essential for glucose uptake from the bloodstream. In the absence of insulin, GLUT4 is sequestered in intracellular vesicles. Upon insulin stimulation and Akt activation, these vesicles fuse with the cell membrane, increasing the number of GLUT4 transporters available to move glucose into the cell [7,18]. This process significantly enhances glucose uptake and reduces blood glucose levels.

In GD, the insulin signaling pathway undergoes a series of disruptions, each contributing to the impairment of glucose uptake and the development of insulin resistance. The initial disruption begins with the aberrant phosphorylation of IRS-1 and IRS-2 [7,19,20]. In a healthy state, these proteins are phosphorylated on tyrosine residues, a modification essential for their role in insulin signaling. However, in GD, elevated levels of hormones such as human placental lactogen (hPL) and cortisol, along with pro-inflammatory cytokines like TNF-α and IL-6, promote the phosphorylation of IRS proteins on serine residues instead (Figure 1B). This serine phosphorylation inhibits their function, preventing the proper activation of downstream signaling molecules.

As a result of this impaired IRS function, the activation of PI3K is significantly reduced. This reduction leads to a decrease in PIP3 production and subsequently hampers the recruitment and activation of Akt at the cell membrane. Without adequate activation of Akt, GLUT4 vesicles remain sequestered within the cell and do not translocate to the membrane. This lack of GLUT4 translocation results in reduced glucose uptake by the cells, leading to elevated blood glucose levels [17,18,19,21,22].

In GD, mitochondrial efficiency in producing adenosine triphosphate (ATP) is compromised, leading to a decline in cellular energy levels. Concurrently, dysfunctional mitochondria generate excessive reactive oxygen species (ROS). Elevated ROS levels induce oxidative stress, disrupting the balance between ROS production and antioxidant defenses [23] and promoting the serine phosphorylation of IRS-1 [24,25,26]. This oxidative stress not only disrupts insulin signaling but also damages cellular components such as lipids, proteins, and DNA, further impairing metabolic regulation. The systemic effects of these disruptions are profound. In skeletal muscle, the impaired glucose uptake leads to decreased glycogen storage and reduced overall glucose clearance from the bloodstream. In adipose tissue, disrupted lipid metabolism increases the release of free fatty acids. These free fatty acids can interfere with insulin signaling in other tissues, such as the liver, creating a feedback loop that worsens insulin resistance [27].

### 2.2. Hormonal Influences on Insulin Resistance

**Figure 2:**
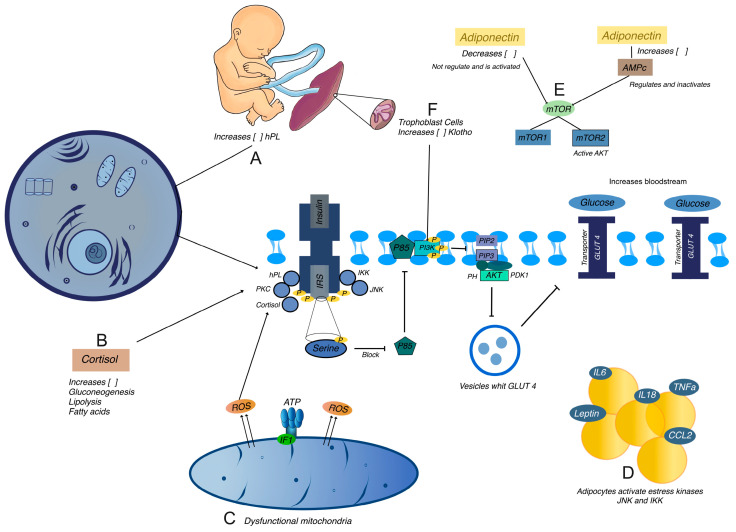
Molecular and hormonal pathways contributing to insulin resistance in gestational diabetes. (**A**) Produced by the placenta, Human Placental Lactogen (hPL) levels increase during pregnancy, promoting lipolysis and reducing maternal glucose uptake by inhibiting insulin action. This ensures glucose availability for the fetus but also contributes to insulin resistance by inducing serine phosphorylation of IRS-1, disrupting downstream signaling pathways essential for glucose uptake. (**B**) Elevated cortisol levels in GD enhance gluconeogenesis and lipolysis, increasing blood glucose and free fatty acids. These free fatty acids activate Protein Kinase C (PKC), which further promotes serine phosphorylation of IRS-1, exacerbating insulin resistance. (**C**) Dysfunctional mitochondria in GD produce excess reactive oxygen species (ROS), leading to oxidative stress. This oxidative damage further impairs insulin signaling by promoting aberrant serine phosphorylation of IRS-1, contributing to metabolic disturbances. (**D**) Cytokines such as TNF-α, IL-6, IL-18, and leptin, secreted by adipocytes and immune cells, activate stress kinases (JNK and IKK). These kinases further inhibit insulin signaling by promoting serine phosphorylation of IRS-1, linking chronic inflammation with impaired glucose uptake. (**E**) Overactivation of the mTOR pathway in GD, partly due to reduced adiponectin levels, leads to increased serine phosphorylation of IRS-1, disrupting insulin signaling. This pathway also impacts cellular growth and autophagy, exacerbating metabolic dysregulation. (**F**) Klotho, a protein overexpressed in trophoblastic cells in GD, interferes with normal insulin signaling by promoting serine phosphorylation of IRS-1. This disruption reduces PI3K and Akt activation, impairing glucose uptake and contributing to hyperglycemia.

GD is driven by complex hormonal, inflammatory, and metabolic mechanisms that impair insulin signaling and glucose regulation (Figure 2).

#### 2.2.1. Human Placental Lactogen (hPL)

hPL, a hormone produced by the placenta, modulates the maternal metabolic state to ensure an adequate energy supply for the growing fetus. As pregnancy progresses, hPL levels increase steadily. One of the primary functions of hPL is to promote lipolysis, the breakdown of fat stores into free fatty acids. These free fatty acids act as an alternative energy source for the mother, thus preserving glucose for the fetus. Additionally, hPL reduces maternal glucose uptake by inhibiting the action of insulin [28]. This ensures that a consistent supply of glucose is available to the fetus, meeting its energy needs.

In GD, the levels of hPL are significantly elevated, which exacerbates insulin resistance. The elevated hPL levels interfere with the normal insulin signaling pathways. Specifically, hPL increases the phosphorylation of IRS-1 on serine residues rather than the usual tyrosine residues. This aberrant phosphorylation of IRS-1 inhibits its ability to activate downstream signaling molecules such as PI3K. Without proper activation of PI3K, the pathway leading to glucose uptake is impaired, resulting in reduced glucose entry into the cells [7,19,29].

#### 2.2.2. Cortisol

Cortisol enhances gluconeogenesis, the process by which glucose is synthesized from non-carbohydrate sources, and increases lipolysis, raising blood glucose levels and the concentration of free fatty acids in the bloodstream. Higher cortisol levels are involved in insulin resistance and the pathogenesis of GD through excessive release of free fatty acids. These free fatty acids can activate protein kinase C (PKC). PKC, in turn, phosphorylates IRS-1 on serine residues, like the effect of hPL. This serine phosphorylation of IRS-1 inhibits its ability to propagate the insulin signal effectively, leading to further impairment of glucose uptake and exacerbation of insulin resistance [7,30].

#### 2.2.3. Inflammatory Pathways and Insulin Resistance

GD is characterized by a state of low-grade chronic inflammation, which significantly contributes to the development and exacerbation of insulin resistance, especially in pregnant women with obesity. Adipose tissue acts as an active endocrine organ in these women, secreting pro-inflammatory cytokines like TNF-α and IL-6, which contribute to metabolic dysfunction. TNF-α, produced by macrophages infiltrating adipose tissue, and IL-6, secreted by both adipocytes and immune cells within the adipose tissue, have local effects on adipose tissue and systemic effects on other tissues, including the liver, muscle, and placenta [31].

Elevated levels of TNF-α and IL-6 in GD activate several stress kinases, including c-Jun N-terminal kinase (JNK) and IκB kinase (IKK). These kinases phosphorylate IRS-1 on serine residues, inhibiting its normal function and preventing it from effectively activating downstream signaling molecules such as PI3K. As a result, the insulin signaling pathway is disrupted, leading to impaired glucose uptake by cells. This serine phosphorylation of IRS-1 by JNK and IKK is a critical molecular event linking inflammation to insulin resistance. When IRS-1 is phosphorylated on serine residues, its ability to interact with the insulin receptor and subsequent downstream signaling components is diminished, hampering the translocation of GLUT4 to the cell membrane and reducing glucose uptake from the bloodstream into the cells [16,19,32]. Consequently, blood glucose levels remain elevated, contributing to hyperglycemia observed in GD.

The chronic inflammatory state in GD extends beyond adipose tissue, affecting multiple organs and tissues, further complicating insulin signaling and metabolic regulation. Inflammatory cytokines can alter hepatic glucose production, leading to increased glucose output from the liver, and impair glucose uptake in muscle tissue, adding to overall insulin resistance. The inflammatory environment in GD can also affect placental function, contributing to further metabolic dysregulation and adverse pregnancy outcomes. This systemic inflammation creates a feedback loop that perpetuates the inflammatory state, maintaining the activation of stress kinases like JNK and IKK, leading to persistent serine phosphorylation of IRS-1 and ongoing disruption of insulin signaling [19,33,34].

#### 2.2.4. Mitochondrial Dysfunction and Oxidative Stress

Mitochondrial dysfunction is a pivotal factor in the pathophysiology of GD, significantly impacting skeletal muscle and adipose tissue. Mitochondria are essential organelles responsible for producing cellular energy in the form of ATP through oxidative phosphorylation [24,35]. In GD, mitochondrial function is markedly impaired, leading to a cascade of metabolic disruptions contributing to insulin resistance.

In healthy individuals, mitochondria efficiently generate ATP, which is crucial for numerous cellular processes, including muscle contraction, metabolic reactions, and maintaining cellular homeostasis. However, in GD, mitochondrial dysfunction results in decreased ATP production, limiting the cells’ ability to perform essential functions, particularly in insulin-sensitive tissues like skeletal muscle and adipose tissue. This energy deficiency affects the metabolic capacities of these tissues, contributing to the overall insulin resistance observed in GD [35,36,37].

In addition to reduced ATP production, mitochondrial dysfunction in GD leads to an increased production of ROS, which induces oxidative stress, causing several effects on cellular functions [23,24,38].

One of the critical impacts of elevated ROS levels is the promotion of serine phosphorylation of IRS-1. However, in GD, the increased serine phosphorylation of IRS-1 inhibits its function, preventing it from effectively transmitting insulin signals. This disruption in insulin signaling impairs glucose uptake by cells, exacerbating insulin resistance [7,16,39].

In addition to impairing cellular functions and metabolic regulation, oxidative stress can also disrupt mitochondrial DNA, proteins, and lipids, leading to further mitochondrial damage and dysfunction [38,39,40]. This creates a vicious cycle where oxidative stress exacerbates mitochondrial dysfunction, leading to increased ROS production and further metabolic disturbances.

The broader impact of mitochondrial dysfunction in GD extends beyond insulin signaling. Impaired mitochondrial function can disrupt the entire cellular metabolic network. For instance, incomplete metabolic intermediates can accumulate, further disturbing metabolic homeostasis. Additionally, mitochondrial dysfunction can lead to increased apoptosis or necrosis of cells, which can further impair tissue function and contribute to insulin resistance [41,42].

In skeletal muscle, mitochondrial dysfunction impairs the muscle’s ability to effectively utilize glucose, reducing glucose uptake and storage as glycogen. This impairment decreases the muscle’s capacity to remove glucose from the bloodstream, contributing to hyperglycemia. In adipose tissue, mitochondrial dysfunction disrupts lipid metabolism, leading to increased lipolysis and the release of free fatty acids into the circulation. These free fatty acids can further impair insulin signaling in other tissues, such as the liver, creating a systemic effect that worsens insulin resistance [7,30,37].

#### 2.2.5. mTOR Pathway Dysregulation

In the intricate balance of cellular metabolism, the mTOR pathway stands as a critical regulator. This pathway acts as a nutrient sensor, orchestrating cell growth, metabolism, and proliferation by integrating signals from nutrients, energy levels, and growth factors. Under normal conditions, mTOR ensures that cells appropriately respond to nutrient availability, promoting growth and maintaining metabolic homeostasis [43,44].

However, in the context of GD, the mTOR pathway becomes abnormally activated, leading to significant metabolic disturbances. This heightened activation is closely linked to the aberrant serine phosphorylation of IRS-1, a key player in the insulin signaling cascade [7,16,45].

In GD, this finely tuned process is disrupted. Elevated serine phosphorylation of IRS-1, driven by the overactive mTOR pathway, impairs the ability of IRS-1 to activate PI3K. This disruption reduces PIP3 production, subsequently impairing Akt activation. Without adequate activation of Akt, the translocation of GLUT4 to the cell membrane is hindered, resulting in decreased glucose uptake. Consequently, glucose accumulates in the bloodstream, contributing to hyperglycemia and insulin resistance [16,46].

#### 2.2.6. Role of Adiponectin

Adiponectin is an adipokine mainly secreted by adipose tissue. It has anti-inflammatory effects and participates in insulin sensitization in different tissues through its specific receptors, AdipR1 and AdipoR2. AdipoR1 participates in the activation of the AMP-activated protein kinase (AMPK) pathway, promoting fatty acid oxidation and facilitating glucose uptake, whereas AdipoR2 regulates metabolic homeostasis by activating the peroxisome proliferator-activated receptor alpha (PPAR-α) pathway. These mechanisms in normal physiologic conditions allow an adequate insulin response [47]. In GD, the inflammatory environment caused by cytokines such as TNF-α and IL-6 negatively impacts adiponectin levels, impairing AdipoR1 and AdipoR2 signaling and affecting AMPK activation. This leads to lower fatty acid oxidation and reduced glucose uptake, exacerbating insulin resistance.

Interestingly, a critical regulator of the mTOR pathway is adiponectin through AMPK, which in turn inhibits mTOR activity. This inhibition is essential for preventing excessive cell growth and maintaining insulin sensitivity [47,48]. However, in individuals with GD, adiponectin levels are typically lower. This reduction in adiponectin leads to decreased activation of AMPK, diminishing its inhibitory effect on mTOR. As a result, mTOR activity becomes unchecked, promoting persistent activation of the pathway [49,50,51].

The persistent activation of mTOR exacerbates metabolic dysregulation. It not only enhances the serine phosphorylation of IRS-1, further impairing insulin signaling, but also promotes anabolic processes such as protein synthesis and cell growth [7,49]. Moreover, heightened mTOR activity reduces autophagy, a process essential for cellular cleanup and maintenance. Autophagy helps remove damaged cellular components and supports metabolic homeostasis. Reduced autophagy due to increased mTOR activity leads to the accumulation of cellular damage and metabolic waste, further exacerbating metabolic disturbances [7,16,45].

#### 2.2.7. Klotho Overexpression and Insulin Resistance

Recent studies have elucidated the role of Klotho, a protein expressed in various tissues, including the placenta, in exacerbating insulin resistance, particularly in GD [52,53]. The overexpression of Klotho in trophoblastic cells significantly interferes with the insulin signaling pathway, a process critical for maintaining glucose homeostasis [53].

Klotho interferes with the normal phosphorylation of IRS-1 by promoting its phosphorylation on serine residues instead of tyrosine residues. This aberrant phosphorylation significantly hampers the ability of IRS-1 to activate PI3K. Consequently, the production of PIP3 is diminished, which in turn reduces the recruitment and activation of Akt. Without sufficient activation of Akt, the translocation of GLUT4 to the cell membrane is impaired, leading to decreased glucose uptake by cells [53,54]. The consequences of Klotho overexpression extend beyond impaired glucose uptake. The disrupted insulin signaling leads to hyperglycemia. Elevated blood glucose levels not only exacerbate insulin resistance but also induce glucotoxicity, damaging insulin signaling pathways further [53]. This creates a feedback loop where insulin resistance worsens, leading to increased blood glucose levels, oxidative stress, and inflammation—factors already heightened in GD [17,53,55]. These metabolic disturbances have broader implications for pregnancy outcomes. Persistent insulin resistance and hyperglycemia contribute to placental dysfunction, increasing the risk of pregnancy complications such as intrauterine growth restriction, fetal macrosomia, and preeclampsia. Additionally, the long-term effects of insulin resistance and glucotoxicity can predispose both the mother and the fetus to type 2 diabetes and cardiovascular diseases later in life.

#### 2.2.8. Mitochondrial Dysfunction in Trophoblastic Cells

Mitochondria are essential organelles responsible for generating ATP, the cell’s primary energy currency, through the process of oxidative phosphorylation. In GD, the function of mitochondria in trophoblastic cells is markedly impaired, leading to a cascade of metabolic disturbances that further aggravate insulin resistance [56].

Under normal conditions, mitochondria efficiently produce ATP, which is crucial for a wide range of cellular functions, including metabolic reactions, cellular maintenance, and energy-intensive processes such as muscle contraction. However, in GD, mitochondrial dysfunction leads to a significant reduction in ATP production. This energy deficit hampers the ability of trophoblastic cells to perform essential metabolic functions, contributing to an overall decrease in cellular efficiency and vitality [57,58].

Concurrently, dysfunctional mitochondria produce elevated levels of ROS, which are harmful byproducts of cellular metabolism. Under normal conditions, cells maintain a delicate balance between ROS production and antioxidant defenses. However, in GD, the increased production of ROS overwhelms the cellular antioxidant systems, leading to a state of oxidative stress. Elevated ROS levels cause oxidative damage to various cellular components, including lipids, proteins, and DNA. This damage disrupts normal cellular functions and further impairs metabolic processes [24,59].

One of the critical impacts of oxidative stress in GD is the promotion of serine phosphorylation of IRS-1. Normally, insulin signaling involves the phosphorylation of IRS-1 on tyrosine residues, which is necessary for activating downstream signaling pathways that facilitate glucose uptake. However, oxidative stress induced by elevated ROS levels promotes the aberrant phosphorylation of IRS-1 on serine residues. This serine phosphorylation inhibits the normal function of IRS-1, preventing it from effectively transmitting insulin signals. As a result, the insulin signaling pathway is disrupted, leading to impaired glucose uptake by cells and exacerbating insulin resistance [7,24].

The broader implications of mitochondrial dysfunction in GD extend beyond impaired insulin signaling. The accumulation of oxidative damage can initiate a vicious cycle, where oxidative stress further damages mitochondria, exacerbating their dysfunction and increasing ROS production [60]. This cycle amplifies the metabolic disturbances and insulin resistance observed in GD.

Moreover, mitochondrial dysfunction in trophoblastic cells can disrupt the entire cellular metabolic network. For instance, impaired mitochondrial function can lead to the accumulation of incomplete metabolic intermediates, further disturbing metabolic homeostasis. Additionally, mitochondrial dysfunction can trigger apoptotic pathways, leading to cell death and further impairing tissue function [61,62].

### 2.3. Gut Microbiota and Metabolic Regulation

#### 2.3.1. Dysbiosis

**Figure 3:**
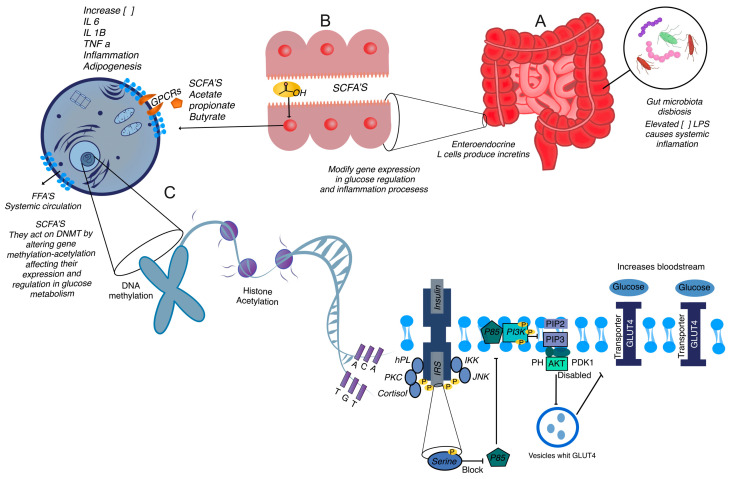
Gut microbiota dysbiosis and its role in metabolic regulation and insulin resistance in gestational diabetes (GD). (**A**) Dysbiosis leads to reduced production of short-chain fatty acids (SCFAs) like acetate, propionate, and butyrate. These SCFAs, produced through microbial fermentation of dietary fibers, interact with G-protein-coupled receptors (GPCRs) and free fatty acid receptors (FFARs) to regulate gene expression linked to glucose metabolism and inflammation. Reduced SCFA levels impair insulin sensitivity and contribute to systemic inflammation, exacerbating GD. (**B**) Dysbiosis increases the production of lipopolysaccharides (LPS) from Gram-negative bacteria, which enter the bloodstream and trigger systemic inflammation. This inflammatory response worsens insulin resistance, a hallmark of GD, through activation of inflammatory cytokines. (**C**) SCFAs also influence epigenetic modifications, including DNA methylation and histone acetylation. These processes regulate the expression of genes involved in glucose metabolism and inflammation. For example, butyrate inhibits histone deacetylases (HDACs), promoting histone acetylation, which enhances the transcription of genes that improve insulin sensitivity and reduce inflammation. SCFAs can also modulate DNA methylation through their impact on DNA methyltransferases (DNMTs), affecting gene activity critical for metabolic regulation in GD.

The gut microbiota, a diverse community of microorganisms residing in the intestines, plays a pivotal role in the metabolism and regulation of GD. Alterations in the composition of the gut microbiota can profoundly impact cellular signaling and metabolic pathways, contributing to the pathophysiology of GD. In GD, there is often a state of dysbiosis, characterized by an imbalance in the microbial community within the gut. This imbalance can result from various factors, including diet, hormonal changes, and genetic predispositions. Dysbiosis has been closely linked to insulin resistance and metabolic dysfunction, as the gut microbiota could act as an immunomodulator. The intestinal microbiota and the metabolites they produce may contribute to the development of insulin resistance in GD, possibly by triggering an inflammatory response or meta-inflammation, which links low-grade inflammation to metabolic disorders [63,64,65] (Figure 3).

#### 2.3.2. Short-Chain Fatty Acids (SCFAs)

The gut microbiota produces a variety of metabolites that can modulate host metabolic pathways. One of the most significant groups of these metabolites is SCFAs, such as acetate, propionate, and butyrate. These SCFAs are produced through the fermentation of dietary fibers by specific bacterial species in the gut. Once produced, SCFAs interact with host receptors, such as G-protein-coupled receptors (GPCRs) and free fatty acid receptors (FFARs), influencing the expression of genes involved in glucose metabolism. SCFAs have been shown to enhance insulin sensitivity and promote glucose homeostasis. For instance, butyrate can stimulate the release of incretin hormones, which enhance insulin secretion in response to meals. Additionally, SCFAs can modulate the inflammatory response, reducing the secretion of pro-inflammatory cytokines that are known to impair insulin signaling. In GD, the altered microbiota composition can lead to changes in the production and profile of these metabolites, thereby affecting insulin sensitivity and glucose regulation [64,65,66].

#### 2.3.3. Other Microbial Metabolites

Beyond SCFAs, other microbial metabolites and components, such as lipopolysaccharides (LPS) from the cell walls of Gram-negative bacteria, can influence host metabolism. Elevated levels of LPS in the bloodstream, a condition known as metabolic endotoxemia, can trigger systemic inflammation. This inflammation can exacerbate insulin resistance, further complicating the metabolic landscape of GD. The interaction between gut microbiota and host metabolism is complex and involves multiple signaling pathways and regulatory mechanisms. The role of the gut microbiota in GD highlights the complex interplay between hormonal, genetic, and microbial factors in regulating metabolism during gestation. Hormonal changes during pregnancy can influence the composition and function of the gut microbiota, while genetic predispositions can affect how the host responds to microbial signals [66,67,68]. This intricate network of interactions underscores the importance of maintaining a balanced gut microbiota for metabolic health during pregnancy.

#### 2.3.4. Epigenetic Modifications

Gene expression and epigenetic modifications play a critical role in the pathogenesis of GD. These modifications, such as DNA methylation and histone modifications, are key regulators of genes involved in metabolism and inflammation. Epigenetic changes can influence gene activity without altering the DNA sequence, affecting how cells respond to metabolic signals [69].

The gut microbiota plays a crucial role in gene expression by producing various metabolites that act as epigenetic signals [70]. One of the most well-studied groups of microbial metabolites are SCFAs. Once produced, SCFAs can enter the host’s circulatory system and interact with various cells and tissues, exerting their effects on gene expression through histone acetylation and DNA methylation [71]. Histone acetylation typically involves the addition of acetyl groups to histone proteins, which can alter the structure of chromatin and make DNA more accessible for transcription. This process can upregulate the expression of genes that are essential for glucose metabolism and anti-inflammatory responses. For example, butyrate, a potent SCFA, is known to act as a histone deacetylase (HDAC) inhibitor. By inhibiting HDACs, butyrate promotes histone acetylation, thereby enhancing the expression of genes involved in maintaining glucose homeostasis and reducing inflammation [72].

#### 2.3.5. DNA Methylation

DNA methylation, on the other hand, generally involves the addition of methyl groups to the cytosine bases in DNA, which often leads to gene silencing. SCFAs can also influence DNA methylation patterns, either directly or indirectly. For instance, butyrate and propionate have been shown to impact the activity of DNA methyltransferases (DNMTs), enzymes responsible for adding methyl groups to DNA. Through modulation of DNMT activity, SCFAs can modify the methylation status of genes that regulate glucose metabolism and inflammatory pathways, thus impacting their expression.

In the context of GD, these epigenetic changes can have profound effects. The altered expression of genes involved in insulin signaling, glucose transport, and inflammatory responses can contribute to the development and exacerbation of insulin resistance and hyperglycemia, hallmarks of GD. For example, genes that encode glucose transporters, insulin receptors, and inflammatory cytokines can be epigenetically modified by SCFAs, leading to either upregulation or downregulation of these critical components in glucose and inflammatory pathways [73,74,75].

Moreover, the effects of metabolites produced by the gut microbiota SCFAs on epigenetic modifications extend beyond immediate metabolic regulation. These changes can also have long-term effects on the health of both the mother and the fetus. Epigenetic modifications induced by SCFAs can be stable and heritable, potentially affecting gene expression patterns in the developing fetus and influencing susceptibility to metabolic diseases later in life [7,45].

### 2.4. Ferroptosis

#### 2.4.1. Mechanism of Ferroptosis

**Figure 4:**
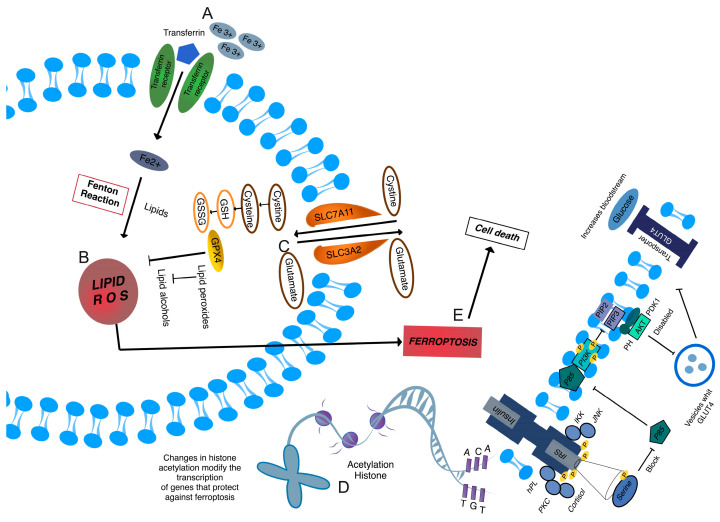
Mechanisms of ferroptosis and its role in insulin resistance in gestational diabetes (GD). (**A**) Free iron (Fe^2+^), resulting from the transport of Fe^3+^ by transferrin, catalyzes the Fenton reaction, which produces reactive oxygen species (ROS). These ROS lead to the peroxidation of lipids, primarily polyunsaturated fatty acids, in the cell membrane. (**B**) Lipid peroxides (Lipid ROS) form as a result of oxidative stress, damaging the cell membrane and triggering ferroptosis. This process is accelerated in GD due to increased oxidative stress. (**C**) The SLC7A11 (xCT) transport system imports cystine, which is necessary for the synthesis of glutathione (GSH). Glutathione peroxidase 4 (GPX4) uses GSH to neutralize lipid peroxides, preventing ferroptosis. A deficiency or inhibition of this system increases susceptibility to ferroptosis. ACSL4 and LPCAT3 facilitate the incorporation of polyunsaturated fatty acids into membrane phospholipids, making them substrates for lipid peroxidation. NRF2 activation increases the expression of antioxidant genes, protecting cells from ferroptosis. (**D**) Changes in DNA methylation and histone acetylation influence the expression of key ferroptosis-related genes. For example, histone acetylation at antioxidant gene promoters enhances their expression, providing protection against oxidative stress. However, in GD, these epigenetic modifications can lead to decreased expression of protective genes like GPX4. (**E**) The accumulation of lipid ROS and the failure of antioxidant systems lead to ferroptosis, resulting in cell death. Dying cells release damage-associated molecular patterns (DAMPs), perpetuating inflammation and contributing to the chronic inflammatory state characteristic of GD.

Ferroptosis is a form of programmed cell death distinct from other processes such as apoptosis, necrosis, and autophagy. This type of cell death is primarily regulated by iron accumulation and lipid peroxidation, leading to the degradation of cell membranes and eventual cell death. Recently, ferroptosis has been implicated in various metabolic diseases, including GD. Free iron catalyzes the formation of ROS through the Fenton reaction, leading to the peroxidation of polyunsaturated fatty acids in cell membranes. The resulting lipid peroxides are highly toxic and cause severe damage to cell membranes, resulting in cell death [76] (Figure 4).

#### 2.4.2. Ferroptosis Genetic Regulation

This process is strictly regulated by several genes, among which SLC7A11 (xCT) and GPX4 are prominent. The xCT-GPX4 antioxidant system plays a crucial role in protecting against ferroptosis. SLC7A11 is part of the cystine/glutamate transport system, crucial for the synthesis of glutathione (GSH), a vital antioxidant. GPX4, a GSH-dependent enzyme, reduces lipid hydroperoxides to their non-toxic forms, thereby preventing lipid peroxidation [77]. ACSL4 and LPCAT3 are other enzymes involved in the synthesis of polyunsaturated lipids, which are substrates for peroxidation. ACSL4 facilitates the esterification of polyunsaturated fatty acids into membrane phospholipids, while LPCAT3 remodels membrane phospholipids to include polyunsaturated fatty acids. ACSL4 and LPCAT function as positive regulators of lipid peroxidation by modulating cellular phospholipids, ultimately promoting ferroptosis induction. NFE2L2 (NRF2) is a transcription factor that regulates the expression of several antioxidant and detoxification genes. Under oxidative stress conditions, NRF2 is activated and promotes the expression of genes that protect against ferroptosis [78].

#### 2.4.3. Epigenetic Modifications

Epigenetic modifications, such as DNA methylation, histone acetylation, and regulation by microRNAs, can significantly influence the expression of ferroptosis-related genes. In the context of GD, these modifications can alter cell viability and contribute to the inflammatory milieu characteristic of this condition. Methylation of promoters of key genes like GPX4 or SLC7A11 can decrease their expression, increasing susceptibility to ferroptosis. Changes in histone acetylation can modify chromatin structure and affect the transcription of genes that protect against ferroptosis. For example, histone acetylation at the promoters of antioxidant genes can facilitate their expression in response to oxidative stress [79]. MicroRNAs are small non-coding RNAs that can regulate gene expression post-transcriptionally. In GD, certain microRNAs may be dysregulated, affecting the expression of genes involved in ferroptosis. For instance, the overexpression of miR-500a-5p has been linked to the repression of GPX4, increasing susceptibility to ferroptosis [80,81].

#### 2.4.4. Inflammatory Impact

Ferroptosis contributes to the chronic inflammation observed in GD. Dying cells release DAMPs (damage-associated molecular patterns), which activate immune and inflammatory responses. This cycle of cell death and inflammation perpetuates the inflammatory environment in tissues affected by GD [76,82].

### 2.5. Inflammation and Oxidative Stress

#### 2.5.1. Chronic Inflammation

Chronic inflammation and oxidative stress are hallmark features of GD, contributing significantly to its pathophysiology (Table 2). In GD, inflammation is characterized by elevated levels of pro-inflammatory cytokines such as tumor necrosis factor-alpha (TNF-α) and interleukin-6 (IL-6). These cytokines interfere with insulin signaling pathways, leading to exacerbated insulin resistance [83]. Elevated TNF-α levels inhibit IRS-1 phosphorylation, impairing insulin signaling and reducing glucose uptake in peripheral tissues. IL-6 can induce hepatic insulin resistance by enhancing gluconeogenesis and glycogenolysis [84]. The combined effect of these cytokines perpetuates a state of chronic low-grade inflammation, further exacerbating metabolic dysfunction in GD [24].

#### 2.5.2. Role of Ferroptosis

Ferroptosis also plays a role in inflammation and oxidative stress. The excessive accumulation of iron in cells catalyzes the production of ROS through Fenton reactions, leading to lipid peroxidation and subsequent cell damage. Lipid peroxides, in turn, serve as potent pro-inflammatory mediators, triggering the release of additional cytokines and perpetuating the inflammatory response. The increased ROS production not only contributes to cellular damage but also activates various signaling pathways, such as nuclear factor kappa B (NF-κB), which further amplifies the inflammatory response [85]. For instance, Zhao et al. demonstrated that iron overload and lipid peroxidation in cells could initiate inflammatory cascades, thereby increasing ROS production and contributing to the overall cellular damage observed in GD [76].

#### 2.5.3. Mitochondrial Dysfunction

Mitochondrial dysfunction is another critical factor linking oxidative stress and inflammation in GD. Trophoblastic cells, which play a vital role in placental function, are particularly susceptible to oxidative stress. Mitochondrial dysfunction in these cells leads to increased ROS production, causing oxidative damage to mitochondrial DNA (mtDNA). This damage impairs mitochondrial function, leading to a vicious cycle of increased ROS production and further mitochondrial damage. The impaired mitochondrial function also affects the energy metabolism of trophoblastic cells, exacerbating cellular dysfunction and contributing to placental insufficiency [86,87]. Fisher et al. highlighted that mitochondrial dysfunction in trophoblastic cells is a significant source of ROS in GD, leading to oxidative stress and mtDNA damage, which in turn exacerbate cellular and placental dysfunction [88].

#### 2.5.4. Gut Microbiota

The gut microbiota has also been recognized as a significant player in the regulation of inflammation and oxidative stress in GD. The gut microbiota is composed of trillions of microorganisms that produce various metabolites influencing host metabolism and immune responses. Dysbiosis, or an imbalance in the gut microbiota, has been associated with increased inflammation and oxidative stress. Microbiota-derived metabolites, such as SCFAs and bile acids, can modulate inflammation and oxidative stress through various mechanisms. SCFAs, for example, have anti-inflammatory properties and can enhance the intestinal barrier function, reducing systemic inflammation [68,89,90]. Conversely, an altered gut microbiota composition can lead to increased production of pro-inflammatory metabolites, exacerbating systemic inflammation and oxidative stress. Ye et al. demonstrated that microbiota-derived metabolites could act as modulators of inflammation and oxidative stress, thereby affecting the inflammatory response and redox homeostasis in GD [71].

The SCFAs functioned as energy substances or signaling molecules to interact with the host locally and beyond the gut. Lipopolysaccharide (LPS) contributed to pathophysiology of diseases through activating Toll-like receptor 4 (TLR4) and being involved in inflammatory responses [66].

#### 2.5.5. Interaction with Immune System

Furthermore, the interaction between gut microbiota and the host immune system plays a crucial role in modulating inflammation. The gut-associated lymphoid tissue (GALT) is a key site where the immune system interacts with the gut microbiota. Dysbiosis can lead to an imbalance in immune cell populations and the production of inflammatory cytokines. This altered immune response can spill over into systemic circulation, contributing to the chronic inflammation observed in GD. Additionally, the gut microbiota can influence the production of ROS by host cells. Certain microbial metabolites can enhance the production of ROS, contributing to oxidative stress, while others may have antioxidant properties, helping to maintain redox balance [68,91].

**Table 2:** Inflammatory and oxidative stress mechanisms in gestational diabetes.

Pathophysiological Factor	Key Mechanisms	Impact on Gestational Diabetes (GD)
Chronic Inflammation	Elevated levels of pro-inflammatory cytokines (TNF-α, IL-6).	TNF-α inhibits IRS-1 phosphorylation, reducing insulin signaling and glucose uptake. IL-6 promotes hepatic insulin resistance via gluconeogenesis and glycogenolysis. Chronic low-grade inflammation exacerbates insulin resistance and metabolic dysfunction in GD.
Ferroptosis	Iron accumulation leads to ROS production via Fenton reactions. Lipid peroxidation triggers inflammatory response.	ROS and lipid peroxides amplify inflammation. Activation of NF-κB increases cytokine release, perpetuating inflammatory cascades and cellular damage.
Mitochondrial Dysfunction	Increased ROS production due to mitochondrial damage. Oxidative damage to mitochondrial DNA (mtDNA).	Mitochondrial dysfunction in trophoblastic cells increases ROS levels. Impaired energy metabolism worsens cellular and placental dysfunction, contributing to placental insufficiency and oxidative stress.
Gut Microbiota and Dysbiosis	Dysbiosis leads to altered production of metabolites (SCFAs, bile acids). Modulates immune response and inflammation.	SCFAs have anti-inflammatory effects and enhance intestinal barrier function. Dysbiosis increases pro-inflammatory metabolites, exacerbating systemic inflammation and oxidative stress. Interaction between gut microbiota and immune system regulates inflammation and ROS production.
Interaction with the Immune System	Gut-associated lymphoid tissue (GALT) modulates immune response. Microbial metabolites affect ROS production.	Dysbiosis disrupts immune cell balance and increases inflammatory cytokine production. Microbial metabolites either enhance ROS production (pro-oxidative) or reduce it (antioxidative), influencing redox homeostasis and contributing to chronic inflammation in GD.

TNF-α: Tumor necrosis factor-alpha; IL-6: Interleukin-6; IRS-1: Insulin receptor substrate-1; ROS: Reactive oxygen species; NF-κB: Nuclear factor kappa-light-chain-enhancer of activated B cells; mtDNA: Mitochondrial DNA; SCFAs: Short-chain fatty acids; GALT: Gut-associated lymphoid tissue.

### 2.6. Functions of Placental and Trophoblastic Cells

#### 2.6.1. Placental Function

Placental and trophoblastic cells play an indispensable role in the development and progression of GD (Table 3). These cells are central to the exchange of nutrients and gases between the mother and the fetus, and their dysfunction can have severe consequences for both the mother and the developing fetus. The placenta is a complex organ that facilitates the transfer of oxygen and nutrients from the maternal blood to the fetal blood while also removing waste products from the fetal circulation. Trophoblastic cells, which form the outer layer of the placenta, are particularly crucial in these processes. They invade the maternal endometrium, remodel the maternal spiral arteries, and establish the blood supply necessary for fetal development. Mitochondria are the powerhouses of the cell, responsible for producing ATP through oxidative phosphorylation. In GD, mitochondrial dysfunction leads to reduced ATP production and increased production of ROS [92]. This imbalance between energy production and oxidative stress impairs the ability of trophoblastic cells to perform essential functions, such as endometrial invasion and vascular remodeling, which are critical for placental development. Fisher et al. highlighted that the reduced ATP levels compromise the energy-dependent processes in trophoblastic cells, while the elevated ROS levels cause oxidative damage to cellular components, further impairing cellular function [88].

#### 2.6.2. Angiogenesis

Mitochondrial dysfunction in GD affects the production of key molecules involved in placental angiogenesis, such as soluble fms-like tyrosine kinase-1 (sFlt1) and placental growth factor (PlGF). sFlt1 is a splice variant of the VEGF receptor 1 (VEGFR1) and acts as a potent antagonist of vascular endothelial growth factor (VEGF). PlGF, on the other hand, is a member of the VEGF family that promotes angiogenesis [93]. In GD, an imbalance occurs where sFlt1 levels increase and PlGF levels decrease, disrupting normal angiogenesis [76]. This overexpression of sFlt1 inhibits VEGF activity by preventing its interaction with endothelial receptors, thereby impairing blood vessel formation. Reduced PlGF levels further limit angiogenesis, contributing to inadequate placental vascular development and function [94,95]. The link between this angiogenic impairment and mitochondrial dysfunction stems from the reduced capacity for ATP production and increased oxidative stress. Damaged mitochondria contribute to the overproduction of ROS, which not only aggravates cellular damage but also triggers inflammatory pathways. This chronic inflammation, in turn, exacerbates endothelial dysfunction and disrupts angiogenesis by promoting the overexpression of anti-angiogenic factors like sFlt1.

#### 2.6.3. Insulin Resistance in Trophoblastic Cells

Insulin resistance in trophoblastic cells is a significant factor contributing to the pathophysiology of GD. Insulin resistance refers to the diminished ability of cells to respond to insulin, a hormone that regulates glucose uptake and metabolism. In GD, trophoblastic cells exhibit insulin resistance, which impairs their ability to respond to crucial hormonal signals during pregnancy [96,97]. This insulin resistance is exacerbated by the overexpression of Klotho. Lin et al. demonstrated that elevated Klotho levels in trophoblastic cells contribute to insulin resistance by interfering with insulin signaling pathways [53]. The insulin resistance in trophoblastic cells leads to maternal hyperglycemia and alters the nutrient transfer to the fetus. This dysregulation of nutrient transfer can result in fetal overgrowth or macrosomia, increasing the risk of complications during delivery and metabolic disorders in the offspring [98].

#### 2.6.4. Broader Implications

The dysfunction of placental and trophoblastic cells in GD has broader implications for maternal and fetal health. The impaired invasion of trophoblastic cells into the endometrium and the inadequate remodeling of spiral arteries can lead to placental insufficiency, a condition where the placenta cannot deliver sufficient oxygen and nutrients to the fetus. This insufficiency can result in intrauterine growth restriction, where the fetus is smaller than expected for the gestational age and can increase the risk of stillbirth. The chronic inflammatory state and oxidative stress associated with GD further exacerbate these complications, creating a hostile intrauterine environment that can affect fetal development and long-term health outcomes [99].

**Table 3:** Key roles of placental and trophoblastic cells in gestational diabetes.

Function	Mechanism	Impact on Gestational Diabetes (GD)
Placental Function	Facilitates nutrient and gas exchange between mother and fetus Trophoblastic cells invade the maternal endometrium and remodel spiral arteries	Mitochondrial dysfunction leads to reduced ATP production and increased ROS, impairing placental functions such as nutrient exchange, endometrial invasion, and vascular remodeling. This contributes to placental insufficiency and fetal developmental complications.
Angiogenesis	Involves key molecules: sFlt1 (inhibits VEGF) and PlGF (promotes VEGF-mediated angiogenesis)	Altered expression in GD: Increased sFlt1 inhibits VEGF, while decreased PlGF reduces angiogenesis, leading to endothelial dysfunction, impaired placental vascularization, and poor placental function.
Insulin Resistance in Trophoblastic Cells	Cells have a reduced ability to respond to insulin, affecting glucose metabolism	Insulin resistance in trophoblastic cells, exacerbated by Klotho overexpression, leads to maternal hyperglycemia and disrupted nutrient transfer, contributing to fetal overgrowth (macrosomia) and increased risk of delivery complications.
Broader Implications	Dysfunction of placental and trophoblastic cells affects maternal and fetal health	Impaired trophoblastic invasion and spiral artery remodeling can cause placental insufficiency, leading to IUGR and increased risk of stillbirth. Chronic inflammation and oxidative stress worsen these outcomes.

ATP: Adenosine triphosphate; ROS: Reactive oxygen species; sFlt1: Soluble fms-like tyrosine kinase-1; VEGF: Vascular endothelial growth factor; PlGF: Placental growth factor; IUGR: Intrauterine growth restriction.

## 3. Discussion

### 3.1. Main Findings

Key findings include the disruption of insulin signaling pathways due to aberrant phosphorylation of IRS-1 and IRS-2, resulting in impaired glucose uptake and insulin resistance. Mitochondrial dysfunction in trophoblastic cells leads to reduced ATP production and increased ROS, exacerbating oxidative stress and further impairing insulin signaling. The dysregulation of the mTOR pathway and the overexpression of Klotho protein contribute to persistent hyperglycemia and insulin resistance. Additionally, the role of gut microbiota in modulating inflammation and oxidative stress through metabolites such as SCFAs and bile acids is highlighted. The epigenetic modifications influencing the expression of ferroptosis-related genes and their impact on inflammation and oxidative stress are also discussed. Finally, the dysfunction of placental and trophoblastic cells leading to placental insufficiency and adverse pregnancy outcomes such as IUGR is emphasized.

### 3.2. Clinical Interpretation

The clinical implications of these findings are significant. The impaired insulin signaling and mitochondrial dysfunction observed in GD can directly impact maternal and fetal health by increasing the risk of complications such as preeclampsia, IUGR, and stillbirth. Understanding the mechanisms of insulin resistance in trophoblastic cells and their role in placental dysfunction provides insights into the pathogenesis of GD and its complications. The chronic inflammatory state and oxidative stress associated with GD not only affect maternal glucose metabolism but also create a hostile intrauterine environment that can have long-term effects on fetal development and the risk of metabolic disorders in offspring.

### 3.3. Potential Therapeutic Targets

Modulating insulin signaling pathways through agents that enhance IRS-1 and IRS-2 function or inhibit their aberrant phosphorylation could improve glucose uptake and reduce insulin resistance. Antioxidant therapies that mitigate mitochondrial dysfunction and reduce ROS production may alleviate oxidative stress and improve placental function. Targeting the mTOR pathway and Klotho protein overexpression could also provide novel strategies to manage hyperglycemia and insulin resistance in GD. Furthermore, interventions aimed at restoring gut microbiota balance and enhancing SCFA production could modulate inflammation and improve metabolic outcomes. Epigenetic therapies that modify DNA methylation and histone acetylation patterns may also offer promising approaches to regulate the expression of genes involved in ferroptosis and oxidative stress.

### 3.4. Research and Funding

Given the complex nature of GD, ongoing research is essential to developing more effective prevention and treatment strategies. Increased funding for research into the molecular mechanisms of GD as well as clinical trials for potential therapies is crucial. Public health agencies and policymakers should advocate for increased investment in GD research, which can lead to breakthroughs in understanding and managing the disease. Collaborative efforts between government agencies, healthcare providers, and research institutions can foster an environment of innovation and progress in GD management. Policy initiatives that support research infrastructure and provide grants for GD studies can drive advancements in this field.

### 3.5. Public Health Implications

The insights from this review have significant public health implications, particularly given the increasing prevalence of gestational diabetes worldwide. Understanding the complex pathophysiology of GD can inform public health strategies aimed at early detection, prevention, and management of this condition. For instance, routine screening for biomarkers associated with insulin resistance, oxidative stress, and inflammation could help identify at-risk populations earlier in pregnancy. Additionally, public health initiatives focused on promoting healthy diets and physical activity could mitigate some of the metabolic risk factors associated with GD.

Public health policies that support maternal health, such as providing access to prenatal care and nutritional counseling, can also play a crucial role in reducing the incidence and severity of GD. Moreover, public awareness campaigns to educate women about the risks of GD and the importance of maintaining a healthy lifestyle during pregnancy can empower individuals to make informed health decisions. Integrating these strategies into broader maternal and child health programs can lead to better health outcomes and reduce the long-term burden of GD on healthcare systems. Children born to mothers with GD are at an increased risk of developing metabolic disorders later in life. Therefore, public health policies should include provisions for long-term follow-up and preventive care for these children. For mothers, the risk of developing type 2 diabetes and cardiovascular diseases post-pregnancy underscores the need for continued health monitoring and lifestyle interventions. Integrating postnatal care programs that focus on weight management, glucose monitoring, and cardiovascular health can help reduce these long-term risks.

### 3.6. Global Health Perspective

Gestational diabetes is a global health issue, with varying prevalence and management practices across different regions. International collaboration and knowledge-sharing can help address this disparity. Developing standardized guidelines for GD screening, diagnosis and treatment that can be adapted to different healthcare systems is essential. Global health organizations should prioritize GD as a key area of focus and work towards creating a cohesive strategy to combat its rise. This includes facilitating international research collaborations, sharing best practices, and providing resources for low- and middle-income countries to improve their GD management programs.

## 4. Materials and Methods

### 4.1. Eligibility Criteria

We conducted a systematic search using specific medical subject headings (MeSH) terms related to the cellular and molecular pathophysiology of GD, including “gestational diabetes mellitus”, “cellular mechanisms”, “molecular mechanisms”, “insulin resistance”, “oxidative stress”, “inflammation”, “placental function”, “trophoblast cells”, and “mitochondrial dysfunction”. Detailed descriptions of the search strategy and the specific query syntaxes used can be found in Table S1.

The inclusion criteria for this review were original studies, systematic reviews, and meta-analyses that focused on the impact of GD on maternal and fetal health. This review adhered to the PRISMA guidelines for systematic reviews and meta-analyses (Figure S1).

### 4.2. Study Selection

Abstracts identified as relevant were assessed by two independent evaluators (J.T.T. and Z.A.C.M.). This initial screening was conducted without knowledge of the articles’ authorship, author affiliations, or study results to minimize bias. Articles that appeared relevant based on their titles and abstracts were selected for a more detailed review. In the second stage, the preselected articles were read in their entirety to ensure they met all the inclusion criteria.

### 4.3. Data Extraction and Analysis

Data extraction from the selected articles was performed using a standardized form designed to capture essential study characteristics. This form included details such as the author, year of publication, country, and study design. Additionally, it captured specific details related to maternal and fetal outcomes associated with GD, key findings, and conclusions drawn from each study. The results obtained from the selected articles were then analyzed and organized for presentation in relevant sections of this review. The most significant findings were highlighted, and connections were established between different aspects of GD’s impact on maternal and fetal health. This systematic approach ensured that the review provided a thorough and nuanced understanding of the current state of research on GD and its implications for pregnancy outcomes (Table S2).

## 5. Conclusions

GD is a complex disorder involving disruptions in insulin signaling, mitochondrial dysfunction, chronic inflammation, and oxidative stress, leading to impaired glucose regulation and insulin resistance. Despite progress in understanding these mechanisms, effective prevention and treatment strategies remain limited. Public health efforts must prioritize early detection, preventive measures, and healthy lifestyles to reduce GD prevalence. International collaboration and standardized guidelines are crucial for addressing disparities in GD management and improving maternal and fetal health outcomes. Continued research is essential for developing targeted therapies that enhance outcomes for both mothers and their babies.

## Figures and Tables

**Table 1:** Cellular and molecular pathways involved in the pathophysiology of gestational diabetes (GD).

Pathway	Description	Impact on GD	Key Factors Affected in GD
Insulin Signaling	Insulin binds to its receptor, triggering a phosphorylation cascade involving IRS, PI3K, and Akt, leading to glucose uptake.	Disruption leads to impaired glucose uptake and insulin resistance.	IRS-1/IRS-2 phosphorylation, PI3K, Akt activation, GLUT4 translocation
Mitochondrial Dysfunction	Mitochondria produce ATP and ROS, maintaining cellular energy balance and signaling.	Reduced ATP production and increased ROS cause oxidative stress and further disrupt insulin signaling.	ATP production, ROS generation, cellular oxidative stress
Hormonal Influences	Hormones like hPL and cortisol modulate metabolism, impacting insulin sensitivity and glucose regulation.	Elevated hPL and cortisol levels exacerbate insulin resistance.	hPL, cortisol, IRS-1 phosphorylation
Inflammatory Pathways	Chronic inflammation driven by cytokines affects multiple tissues, impairing insulin signaling and glucose uptake.	Persistent inflammation perpetuates insulin resistance and metabolic dysregulation.	TNF-α, IL-6, JNK, IKK, IRS-1 phosphorylation
mTOR Pathway	Regulates cell growth and metabolism by integrating nutrient and growth factor signals.	Dysregulation leads to abnormal cell growth, reduced autophagy, and exacerbated insulin resistance.	mTOR activation, IRS-1 phosphorylation, PI3K, Akt, autophagy
Klotho Overexpression	Klotho protein modulates various metabolic pathways, influencing insulin signaling.	Overexpression impairs insulin signaling, leading to persistent hyperglycemia and glucotoxicity.	IRS-1 phosphorylation, PI3K, Akt, GLUT4 translocation
Gut Microbiota	Microbial community in the gut influences host metabolism and immune responses.	Dysbiosis affects metabolite production, impacting insulin sensitivity and inflammation.	SCFAs (acetate, propionate, butyrate), LPS, gut-associated lymphoid tissue
Gene Expression and Epigenetics	Epigenetic modifications regulate gene activity, influencing metabolic and inflammatory pathways.	Altered epigenetic regulation contributes to insulin resistance and metabolic dysfunction.	DNA methylation, histone acetylation, SCFAs, gene expression
Ferroptosis	Iron-dependent cell death caused by lipid peroxidation and ROS production.	Contributes to oxidative stress and chronic inflammation, exacerbating GD pathology.	Iron accumulation, lipid peroxidation, ROS, SLC7A11, GPX4
Functions of Placental and Trophoblastic Cells	Cells crucial for nutrient and gas exchange between mother and fetus, impacting fetal development.	Dysfunction leads to placental insufficiency, impaired angiogenesis, and adverse fetal outcomes.	sFlt1, PlGF, insulin resistance, angiogenesis

IRS: Insulin receptor substrate; PI3K: Phosphoinositide 3-kinase; Akt Protein kinase B; GLUT4 Glucose transporter type 4; ATP: Adenosine triphosphate; ROS: Reactive oxygen species; hPL: human placental lactogen; TNF-α: Tumor necrosis factor-alpha; IL-6: Interleukin-6; JNK: c-Jun N-terminal kinase; IKK: IκB kinase; mTOR: Mammalian target of rapamycin; SCFAs: Short-chain fatty acids; LPS: lipopolysaccharides; DNA: Deoxyribonucleic acid; SLC7A11: Solute carrier family 7 member 11; GPX4: Glutathione peroxidase 4; sFlt1: Soluble fms-like tyrosine kinase-1; PlGF: Placental growth factor.

## Data Availability

The data employed for conducting this narrative review are available upon request to the following e-mail: torresmmf@gmail.com.

## Supplementary Materials

The following supporting information can be downloaded at: https://www.mdpi.com/article/10.3390/ijms252111641/s1.
