# Supplementary material for: Cellular and Molecular Pathophysiology of Gestational Diabetes
Source: Int J Mol Sci. 2024 Oct 30;25(21):11641. doi: 10.3390/ijms252111641 (PMC11546748; doi:10.3390/ijms252111641)
Supplement: Supplementary file 1 [file ijms-25-11641-s001.zip › ijms-3233639-supplementary.pdf]

Table S1. Search Strategies

Database	Search Terms
PubMed	("Diabetes, Gestational"[Mesh] OR "gestational diabetes mellitus"[Title/Abstract] OR "GDM"[Title/Abstract]) AND ("Genetics"[Mesh] OR "genetic susceptibility"[Title/Abstract] OR "genetic predisposition"[Title/Abstract] OR "genetic variant"[Title/Abstract] OR "genome-wide association study"[Title/Abstract] OR "GWAS"[Title/Abstract] OR "candidate gene"[Title/Abstract]) AND ("risk factor"[Title/Abstract] OR "association"[Title/Abstract] OR "susceptibility"[Title/Abstract])
MEDLINE	("gestational diabetes mellitus" OR "GDM") AND ("genetic susceptibility" OR "genetic predisposition" OR "genetic variant" OR "genome-wide association study" OR "GWAS" OR "candidate gene") AND ("risk factor" OR "association" OR "susceptibility")
Google Scholar	"gestational diabetes mellitus" AND "genetics" AND ("risk factor" OR "association" OR "susceptibility")
Scopus	("gestational diabetes mellitus" OR "GDM") AND ("genetic susceptibility" OR "genetic predisposition" OR "genetic variant" OR "genome-wide association study" OR "GWAS" OR "candidate gene") AND ("risk factor" OR "association" OR "susceptibility")

Figure S1. PRISMA flow diagram

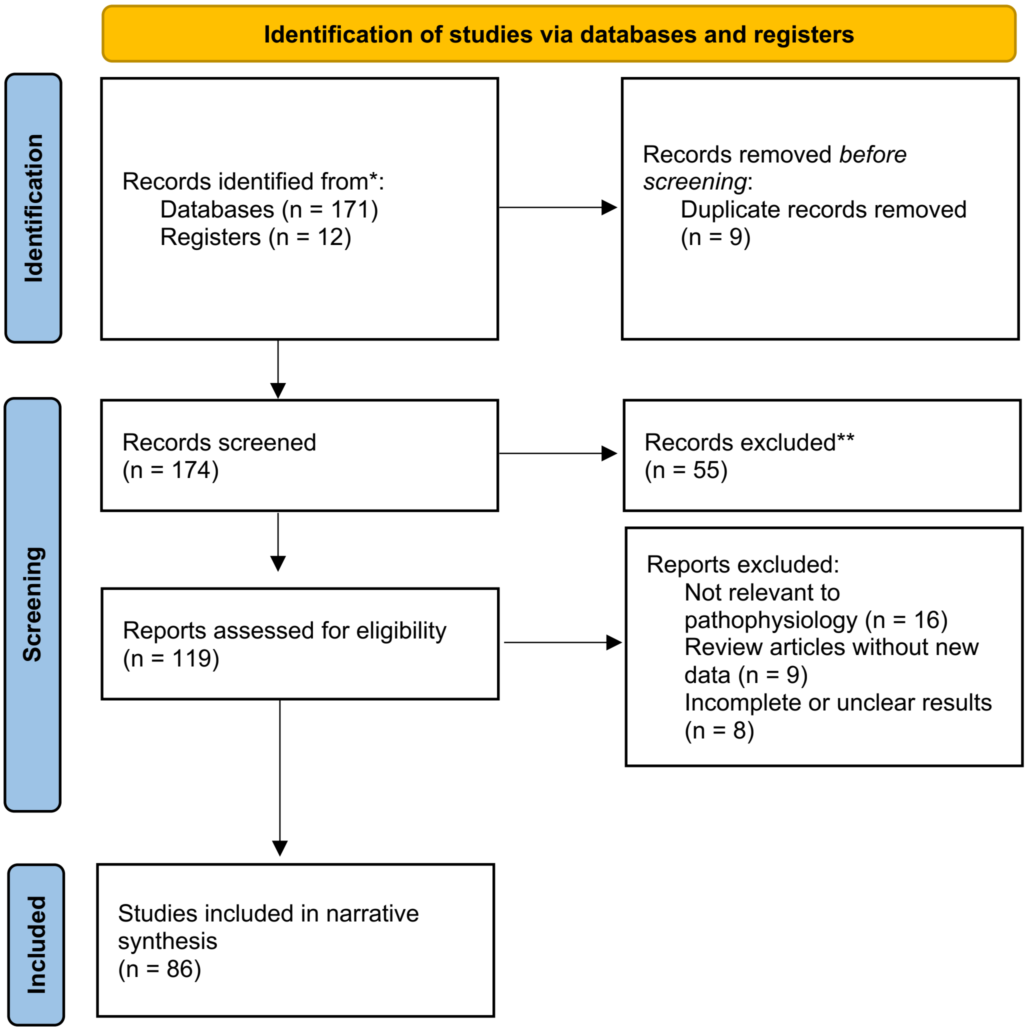

*Consider, if feasible to do so, reporting the number of records identified from each database or register searched (rather than the total number across all databases/registers).

**If automation tools were used, indicate how many records were excluded by a human and how many were excluded by automation tools.

**Table S2.** Summary of included studies					
No.	Title	Authors	Year	Journal	DOI
1	Cellular mechanisms for insulin resistance in normal pregnancy and gestational diabetes	Barbour, L. A.; McCurdy, C. E.; Hernandez, T. L.; Kirwan, J. P.; Catalano, P. M.; Friedman, J. E.	2007	Diabetes care	DOI: 10.2337/dc07-s202
2	Phosphorylation Codes in IRS-1 and IRS-2 Are Associated with the Activation/Inhibition of Insulin Canonical Signaling Pathways	Martínez Báez, A.; Ayala, G.; Pedroza-Saavedra, A.; González-Sánchez, H. M.; Chihu Amparan, L.	2024	Currents issues in molecular biology	DOI: 10.3390/cimb46010041
3	Expression and function of the insulin receptor substrate proteins in cancer.	Mardilovich, K.; Pankratz, S. L.; Shaw, L. M.	2009	Cell communication and signaling	DOI: 10.1186/1478-811x-7-14
4	Molecular mechanisms of insulin resistance: serine phosphorylation of insulin receptor substrate-1 and increased expression of p85alpha: the two sides of a coin	Draznin, B.	2006	Diabetes	DOI: 10.2337/db06-0391
5	Insulin receptor substrate regulation of phosphoinositide 3-kinase	Metz, H. E.; Houghton, A. M.	2011	Journal of the American Association for Cancer research	DOI: 10.1158/1078-0432.Ccr-10-0434
6	Akt activation is required at a late stage of insulin-induced GLUT4 translocation to the plasma membrane.	van Dam, E. M.; Govers, R.; James, D. E.	2005	Molecular Endocrinology	DOI: 10.1210/me.2004-0413
7	The Pathophysiology of Gestational Diabetes Mellitus.	Plows, J. F.; Stanley, J. L.; Baker, P. N.; Reynolds, C. M.; Vickers, M. H.	2018	International Journal of molecular Sciences	DOI: 10.3390/ijms19113342
8	TNF-alpha is a predictor of insulin resistance in human pregnancy	Kirwan, J. P.; Hauguel-De Mouzon, S.; Lepercq, J.; Challier, J. C.; Huston-Presley, L.; Friedman, J. E.;	2002	Diabetes	DOI: 10.2337/diabetes.51.7.2207

		Kalhan, S. C.; Catalano, P. M.			
9	Insulin signalling and GLUT4 trafficking in insulin resistance	van Gerwen, J.; Shun-Shion, A. S.; Fazakerley, D. J.	2023	Biochemical Society transactions	DOI: 10.1042/bst20221066
10	AKT ISOFORMS-AS160-GLUT4: The defining axis of insulin resistance	Sharma, M.; Dey, C. S.	2021	Reviews in endocrine & metabolic disorders	DOI: 10.1007/s11154-021-09652-2
11	Role of Oxidative Stress and Inflammation in Gestational Diabetes Mellitus	Saucedo, R.; Ortega-Camarillo, C.; Ferreira-Hermosillo, A.; Díaz-Velázquez, M. F.; Meixueiro-Calderón, C.; Valencia-Ortega, J.	2023	Antioxidants Basel	DOI: 10.3390/antiox12101812
12	Impact of mitochondrial reactive oxygen species and apoptosis signal-regulating kinase 1 on insulin signaling.	Imoto, K.; Kukidome, D.; Nishikawa, T.; Matsuhisa, T.; Sonoda, K.; Fujisawa, K.; Yano, M.; Motoshima, H.; Taguchi, T.; Tsuruzoe, K.; et al.	2006	Diabetes	DOI: 10.2337/db05-1187
13	Mechanisms underlying skeletal muscle insulin resistance induced by fatty acids: importance of the mitochondrial function	Martins, A. R.; Nachbar, R. T.; Gorjao, R.; Vinolo, M. A.; Festuccia, W. T.; Lambertucci, R. H.; Cury-Boaventura, M. F.; Silveira, L. R.; Curi, R.; Hirabara, S. M.	2012	Lipids in health and disease	DOI: 10.1186/1476-511x-11-30
14	Lactogenic hormones in relation to maternal metabolic health in pregnancy and postpartum: protocol for a systematic review	Rassie, K. L.; Giri, R.; Melder, A.; Joham, A.; Mousa, A.; Teede, H. J.	2022	BMJ open	DOI: 10.1136/bmjopen-2021-055257
15	Phosphatidylinositol 3-kinase redistribution is associated	Shao, J.; Yamashita, H.;	2002	Diabetes	DOI: 10.2337/diabetes.51.1.19

	with skeletal muscle insulin resistance in gestational diabetes mellitus	Qiao, L.; Draznin, B.; Friedman, J. E.			
16	Advances in free fatty acid profiles in gestational diabetes mellitus	Du, H.; Li, D.; Molive, L. M.; Wu, N.	2024	Journal of translational medicine	DOI: 10.1186/s12967-024-04922-4
17	Glucose-6-Phosphate Dehydrogenase Deficiency Improves Insulin Resistance With Reduced Adipose Tissue Inflammation in Obesity	Ham, M.; Choe, S. S.; Shin, K. C.; Choi, G.; Kim, J. W.; Noh, J. R.; Kim, Y. H.; Ryu, J. W.; Yoon, K. H.; Lee, C. H.; et al.	2016	Diabetes	DOI: 10.2337/db16-0060
18	Tumor necrosis factor-alpha induces skeletal muscle insulin resistance in healthy human subjects via inhibition of Akt substrate 160 phosphorylation	Plomgaard, P.; Bouzakri, K.; Krogh-Madsen, R.; Mittendorfer, B.; Zierath, J. R.; Pedersen, B. K.	2005	Diabetes	DOI: 10.2337/diabetes.54.10.2939
19	Nutrition and Metabolic Adaptations in Physiological and Complicated Pregnancy: Focus on Obesity and Gestational Diabetes	Parrettini, S.; Caroli, A.; Torlone, E.	2020	Frontiers in Endocrinology	DOI: 10.3389/fendo.2020.611929
20	The human placenta in gestational diabetes mellitus. The insulin and cytokine network	Desoye, G.; Hauguel-de Mouzon, S.	2007	Diabetes Care	DOI: 10.2337/dc07-s203
21	Gestational diabetes is characterized by reduced mitochondrial protein expression and altered calcium signaling proteins in skeletal muscle	Boyle, K. E.; Hwang, H.; Janssen, R. C.; DeVente, J. M.; Barbour, L. A.; Hernandez, T. L.; Mandarino, L. J.; Lappas, M.; Friedman, J. E.	2014	PLoS one	DOI: 10.1371/journal.pone.0106872
22	Targeting Mitochondria in Diabetes	Krako Jakovljevic, N.; Pavlovic, K.; Jotic, A.; Lalic, K.; Stoiljkovic, M.; Lukic, L.; Milicic, T.; Macesic, M.; Stanarcic Gajovic, J.; Lalic, N. M.	2021	International Journal of molecular Sciences	DOI: 10.3390/ijms22126642

23	Mitochondrial Dysfunction in Obesity and Reproduction	Das, M.; Sauceda, C.; Webster, N. J. G.	2021	Endocrinology	DOI: 10.1210/endocr/bqaa158
24	ROS production by mitochondria: function or dysfunction	Palma, F. R.; Gantner, B. N.; Sakiyama, M. J.; Kayzuka, C.; Shukla, S.; Lacchini, R.; Cunniff, B.; Bonini, M. G.	2024	Oncogene	DOI: 10.1038/s41388-023-02907-z
25	Antioxidants Maintain Cellular Redox Homeostasis by Elimination of Reactive Oxygen Species.	He, L.; He, T.; Farrar, S.; Ji, L.; Liu, T.; Ma, X.	2017	International journal of experimental cellular physiology, biochemistry, and pharmacology.	DOI: 10.1159/000485089
26	Are oxidative stress-activated signaling pathways mediators of insulin resistance and beta-cell dysfunction?	Evans, J. L.; Goldfine, I. D.; Maddux, B. A.; Grodsky, G. M.	2003	Diabetes	DOI: 10.2337/diabetes.52.1.1
27	Interplay of oxidative stress, cellular communication and signaling pathways in cancer.	Iqbal, M. J.; Kabeer, A.; Abbas, Z.; Siddiqui, H. A.; Calina, D.; Sharifi-Rad, J.; Cho, W. C.	2024	Cell communication and signaling	DOI: 10.1186/s12964-023-01398-5
28	Discovery of metabolic biomarkers for gestational diabetes mellitus in a Chinese population.	Lu, W.; Luo, M.; Fang, X.; Zhang, R.; Li, S.; Tang, M.; Yu, X.; Hu, C.	2021	Nutrition & metabolism	DOI: 10.1186/s12986-021-00606-8
29	Increased placental mitochondrial fusion in gestational diabetes mellitus: an adaptive mechanism to optimize feto-placental metabolic homeostasis?	Abbade, J.; Klemetti, M. M.; Farrell, A.; Ermini, L.; Gillmore, T.; Sallais, J.; Tagliaferro, A.; Post, M.; Caniggia, I.	2020	BMJ open diabetes research & care	DOI: 10.1136/bmjdrc-2019-000923
30	Role of mTOR in Glucose and Lipid Metabolism.	Mao, Z.; Zhang, W.	2018	International Journal of molecular Sciences	DOI: 10.3390/ijms19072043

31	The Mammalian target of rapamycin pathway regulates nutrient-sensitive glucose uptake in man.	Krebs, M.; Brunmair, B.; Brehm, A.; Artwohl, M.; Szendroedi, J.; Nowotny, P.; Roth, E.; Fürnsinn, C.; Promintzer, M.; Anderwald, C.; et al.	2007	Diabetes	DOI: 10.2337/db06-1016
32	Serine phosphorylation of insulin receptor substrate-1: a novel target for the reversal of insulin resistance	Sykiotis, G. P.; Papavassiliou, A. G.	2001	Molecular Endocrinology	DOI: 10.1210/mend.15.11.0725
33	PI3K/AKT, MAPK and AMPK signalling: protein kinases in glucose homeostasis.	Schultze, S. M.; Hemmings, B. A.; Niessen, M.; Tschopp, O.	2012	Expert Reviews in molecular medicine	DOI: 10.1017/s1462399411002109
34	Adiponectin signaling and function in insulin target tissues	Ruan, H.; Dong, L. Q.	2016	Journal of molecular cell biology	DOI: 10.1093/jmcb/mjw014
35	Diminished mTOR signaling: a common mode of action for endocrine longevity factors.	Lamming, D. W.	2014	Springer	DOI: 10.1186/2193-1801-3-735
36	AMPK pathway: an emerging target to control diabetes mellitus and its related complications.	Kakoti, B. B.; Alom, S.; Deka, K.; Halder, R. K.	2024	Journal of Diabetes and metabolic disorders	DOI: 10.1007/s40200-024-01420-8
37	Oleuropein alleviates gestational diabetes mellitus by activating AMPK signaling.	Zhang, Z.; Zhao, H.; Wang, A.	2012	Endocrine connections	DOI: 10.1530/ec-20-0466
38	AMPK, insulin resistance, and the metabolic syndrome.	Ruderman, N. B.; Carling, D.; Prentki, M.; Cacicedo, J. M.	2013	The Journal of Clinical Investigation.	DOI: 10.1172/jci67227
39	The interaction between klotho protein and epigenetic alteration in diabetes and treatment options	Hosseininasab, S. S.; Dhiaa, S. M.; Shahrtash, S. A.; Lak, M.; Faghihkhorasani, A.; Mahdi, F.	2024	Journal of Diabetes and metabolic disorders	DOI: 10.1007/s40200-024-01387-6
40	Upregulation of Klotho Aggravates Insulin Resistance in Gestational Diabetes Mellitus Trophoblast Cells.	Lin, L.; Wang, X.; Zhao, W.; Chen, Y.	2022	Genetics Research	DOI: 10.1155/2022/1500768

41	Role of insulin receptor substance-1 modulating PI3K/Akt insulin signaling pathway in Alzheimer's disease.	Zheng, M.; Wang, P.	2021	3 Biotech	DOI: 10.1007/s13205-021-02738-3
42	Regulation of insulin sensitivity by serine/threonine phosphorylation of insulin receptor substrate proteins IRS1 and IRS2.	Copps, K. D.; White, M. F.	2012	Diabetologia	DOI: 10.1007/s00125-012-2644-8
43	Impairment of insulin signaling pathway PI3K/Akt/mTOR and insulin resistance induced AGEs on diabetes mellitus and neurodegenerative diseases: a perspective review.	Ramasubbu, K.; Devi Rajeswari, V.	2023	Molecular and cellular biochemistry	DOI: 10.1007/s11010-022-04587-x
44	Mitochondria and diabetes. An intriguing pathogenetic role.	Newsholme, P.; Gaudel, C.; Krause, M.	2012	Advances in experimental medicine and biology	DOI: 10.1007/978-94-007-2869-1_10
45	Feto-placental endothelial dysfunction in Gestational Diabetes Mellitus under dietary or insulin therapy.	Wang, J. J.; Wang, X.; Li, Q.; Huang, H.; Zheng, Q. L.; Yao, Q.; Zhang, J.	2023	BMC Endocrine Disorders	DOI: 10.1186/s12902-023-01305-6
46	β-cell mitochondria in diabetes mellitus: a missing puzzle piece in the generation of hPSC-derived pancreatic β-cells?	Diane, A.; Al-Shukri, N. A.; Bin Abdul Mu, U. M. R.; Al-Siddiqi, H. H.	2022	Journal of translational medicine	DOI: 10.1186/s12967-022-03327-5
47	The role of oxidative stress in diabetes mellitus-induced vascular endothelial dysfunction.	An, Y.; Xu, B. T.; Wan, S. R.; Ma, X. M.; Long, Y.; Xu, Y.; Jiang, Z. Z.	2023	Cardiovascular Diabetology	DOI: 10.1186/s12933-023-01965-7
48	The impact of oxidative stress-induced mitochondrial dysfunction on diabetic microvascular complications.	Zhang, Z.; Huang, Q.; Zhao, D.; Lian, F.; Li, X.; Qi, W.	2023	Frontiers in Endocrinology	DOI: 10.3389/fendo.2023.1112363
49	Elevated Medium-Chain Acylcarnitines Are Associated With Gestational Diabetes Mellitus and Early Progression to Type 2 Diabetes and Induce Pancreatic β-Cell Dysfunction.	Batchuluun, B.; Al Rijjal, D.; Prentice, K. J.; Eversley, J. A.; Burdett, E.; Mohan, H.; Bhattacharjee, A.; Gunderson, E. P.; Liu, Y.; Wheeler, M. B.	2018	American Diabetes Association	DOI: 10.2337/db17-1150

50	β-Cell death is decreased in women with gestational diabetes mellitus.	Kenna, L. A.; Olsen, J. A.; Spelios, M. G.; Radin, M. S.; Akirav, E. M.	2016	Diabetology & Metabolic Syndrome	DOI: 10.1186/s13098-016-0175-z
51	Decoding the Gut Microbiota-Gestational Diabetes Link: Insights from the Last Seven Years.	Balleza-Alejandri, L. R.; Peña-Durán, E.; Beltrán-Ramírez, A.; Reynoso-Roa, A. S.; Sánchez-Abundis, L. D.; García-Galindo, J. J.; Suárez-Rico, D. O.	2024	Microorganisms	DOI: 10.3390/microorganisms12061070
52	Gestational diabetes is driven by microbiota-induced inflammation months before diagnosis.	Pinto, Y.; Frishman, S.; Turjeman, S.; Eshel, A.; Nuriel-Ohayon, M.; Shrossel, O.; Ziv, O.; Walters, W.; Parsonnet, J.; Ley, C.; et al.	2023	Gut Microbiota	DOI: 10.1136/gutjnl-2022-328406
53	Gut Microbiota and Gestational Diabetes Mellitus: A Review of Host-Gut Microbiota Interactions and Their Therapeutic Potential.	Hasain, Z.; Mokhtar, N. M.; Kamaruddin, N. A.; Mohamed Ismail, N. A.; Razalli, N. H.; Gnanou, J. V.; Raja Ali, R. A.	2020	Frontiers in cellular and infection microbiology	DOI: 10.3389/fcimb.2020.00188
54	Interactions between host and gut microbiota in gestational diabetes mellitus and their impacts on offspring.	Wang, S.; Cui, Z.; Yang, H.	2024	BMC Microbiology	DOI: 10.1186/s12866-024-03255-y
55	Impacts of gut microbiota on gestational diabetes mellitus: a comprehensive review.	Huang, L.; Thonusin, C.; Chattipakorn, N.; Chattipakorn, S. C.	2021	European Journal of nutrition	DOI: 10.1007/s00394-021-02483-6
56	Gestational diabetes is associated with change in the gut microbiota composition in third trimester of pregnancy and postpartum.	Crusell, M. K. W.; Hansen, T. H.; Nielsen, T.; Allin, K. H.; Rühlemann, M. C.; Damm, P.;	2018	Microbiome	DOI: 10.1186/s40168-018-0472-x

		Vestergaard, H.; Rørbye, C.; Jørgensen, N. R.; Christiansen, O. B.; et al.			
57	Integrative metagenomic and metabolomic analyses reveal gut microbiota-derived multiple hits connected to development of gestational diabetes mellitus in humans.	Ye, D.; Huang, J.; Wu, J.; Xie, K.; Gao, X.; Yan, K.; Zhang, P.; Tao, Y.; Li, Y.; Zang, S.; et al.	2023	Gut Microbes	DOI: 10.1080/19490976.2022.2154552
58	Ferroptosis and its potential role in gestational diabetes mellitus: updated evidence from pathogenesis to therapy.	Zhao, Y.; Gao, Q.; Li, B.; Wang, Y.; Wang, Y.	2023	Frontiers in Endocrinology	DOI: 10.3389/fendo.2023.1177547
59	Ferroptosis: an iron-dependent form of nonapoptotic cell death.	Dixon, S. J.; Lemberg, K. M.; Lamprecht, M. R.; Skouta, R.; Zaitsev, E. M.; Gleason, C. E.; Patel, D. N.; Bauer, A. J.; Cantley, A. M.; Yang, W. S.; et al.	2012	Cell Press Journal	DOI: 10.1016/j.cell.2012.03.042
60	FSP1 is a glutathione-independent ferroptosis suppressor	Doll, S.; Freitas, F. P.; Shah, R.; Aldrovandi, M.; da Silva, M. C.; Ingold, I.; Goya Grocin, A.; Xavier da Silva, T. N.; Panzilius, E.; Scheel, C. H.; et al.	2019	Nature	DOI: 10.1038/s41586-019-1707-0
61	The role of maternal DNA methylation in pregnancies complicated by gestational diabetes.	Dias, S.; Willmer, T.; Adam, S.; Pheiffer, C.	2022	Frontiers in clinical diabetes and healthcare.	DOI: 10.3389/fcdhc.2022.982665
62	Exposure to Gestational Diabetes Mellitus (GDM) alters DNA methylation in placenta and fetal cord blood.	Awamleh, Z.; Butcher, D. T.; Hanley, A.; Retnakaran, R.; Haertle, L.; Haaf, T.; Hamilton, J.; Weksberg, R.	2021	Diabetes research and clinical practice	DOI: 10.1016/j.diabres.2021.108690

63	Activation of the p62-Keap1-NRF2 pathway protects against ferroptosis in hepatocellular carcinoma cells.	Sun, X.; Ou, Z.; Chen, R.; Niu, X.; Chen, D.; Kang, R.; Tang, D.	2016	Hepatology	DOI: 10.1002/hep.28251
64	Ferroptosis: A Regulated Cell Death Nexus Linking Metabolism, Redox Biology, and Disease.	Stockwell, B. R.; Friedmann Angeli, J. P.; Bayir, H.; Bush, A. I.; Conrad, M.; Dixon, S. J.; Fulda, S.; Gascón, S.; Hatzios, S. K.; Kagan, V. E.; et al.	2017	Cell	DOI: 10.1016/j.cell.2017.09.021
65	Obesity and pregnancy: mechanisms of short term and long term adverse consequences for mother and child.	Catalano, P. M.; Shankar, K.	2017	TheBMJ	DOI: 10.1136/bmj.j1
66	Endoplasmic reticulum stress is increased in adipose tissue of women with gestational diabetes.	Liong, S.; Lappas, M.	2015	PLOS One	DOI: 10.1371/journal.pone.0122633
67	Ferroptosis: mechanisms, biology and role in disease.	Jiang, X.; Stockwell, B. R.; Conrad, M.	2021	Nat Rev Mol Cell Biol	DOI: 10.1038/s41580-020-00324-8
68	Impact of Obesity and Hyperglycemia on Placental Mitochondria.	Mandò, C.; Anelli, G. M.; Novielli, C.; Panina-Bordignon, P.; Massari, M.; Mazzocco, M. I.; Cetin, I.	2018	Oxidative Medicine and Cellular Longevity	DOI: 10.1155/2018/2378189
69	The role of oxidative stress in the pathophysiology of gestational diabetes mellitus.	Lappas, M.; Hiden, U.; Desoye, G.; Froehlich, J.; Hauguel-de Mouzon, S.; Jawerbaum, A.	2011	Antioxidants & Redox Signaling	DOI: 10.1089/ars.2010.3765
70	Mitochondrial dysfunction in placental trophoblast cells experiencing gestational diabetes mellitus.	Fisher, J. J.; Vanderpeet, C. L.; Bartho, L. A.; McKeating, D. R.; Cuffe, J. S. M.; Holland, O. J.; Perkins, A. V.	2021	The Journal of Physiology	DOI: 10.1113/jp280593

71	Gut microbiome in gestational diabetes: a cross-sectional study of mothers and offspring 5 years postpartum.	Hasan, S.; Aho, V.; Pereira, P.; Paulin, L.; Koivusalo, S. B.; Auvinen, P.; Eriksson, J. G.	2018	Acta Obstetricia et Gynecologica Scandinavica	DOI: 10.1111/aogs.13252
72	The role of short-chain fatty acids in the interplay between diet, gut microbiota, and host energy metabolism.	den Besten, G.; van Eunen, K.; Groen, A. K.; Venema, K.; Reijngoud, D. J.; Bakker, B. M.	2013	Journal of Lipid Research	DOI: 10.1194/jlr.R036012
73	The gut microbiome of obese postpartum women with and without previous gestational diabetes mellitus and the gut microbiota of their babies.	Dualib, P. M.; Fernandes, G.; Taddei, C. R.; Carvalho, C. R. S.; Sparvoli, L. G.; Bittencourt, C.; Silva, I. T.; Mattar, R.; Ferreira, S. R. G.; Dib, S. A.; et al.	2022	Diabetes Research and Clinical Practice	DOI: 10.1186/s13098-022-00954-2
74	The Placental Role in Gestational Diabetes Mellitus: A Molecular Perspective.	Calvo, M. J.; Parra, H.; Santeliz, R.; Bautista, J.; Luzardo, E.; Villasmil, N.; Martínez, M. S.; Chacín, M.; Cano, C.; Checa-Ros, A.; et al.	2024	Touch Endocrinology	DOI: 10.17925/ee.2024.20.1.5
75	Mitochondrial Dysfunction in the Pathogenesis of Preeclampsia.	Hu, X. Q.; Zhang, L.	2022	Currents Hypertension reports	DOI: 10.1007/s11906-022-01184-7
76	Placental Growth Factor and Pregnancy-Associated Plasma Protein-A as Potential Early Predictors of Gestational Diabetes Mellitus.	Yanachkova, V.; Staynova, R.; Stankova, T.; Kamenov, Z.	2023	Medicina Kaunas	DOI: 10.3390/medicina59020398
77	Vascular endothelial growth factor and its receptors regulation in gestational diabetes mellitus and eclampsia.	Bolatai, A.; He, Y.; Wu, N.	2022	Journal of Translational Medicine	DOI: 10.1186/s12967-022-03603-4
78	Maternal Metabolites Associated With Gestational	Liu, Y.; Kuang, A.; Bain, J. R.;	2021	The Journal of Clinical	DOI: 10.1210/clinem/dgab513

	Diabetes Mellitus and a Postpartum Disorder of Glucose Metabolism.	Muehlbauer, M. J.; Ilkayeva, O. R.; Lowe, L. P.; Metzger, B. E.; Newgard, C. B.; Scholtens, D. M.; Lowe, W. L.		Endocrinology & Metabolism	
79	Role of Insulin in Placental Transport of Nutrients in Gestational Diabetes Mellitus.	Ruiz-Palacios, M.; Ruiz-Alcaraz, A. J.; Sanchez-Campillo, M.; Larqué, E.	2017	Annals of Nutrition and Metabolism	DOI: 10.1159/000455904
80	Fetal programming and gestational diabetes mellitus.	Monteiro, L. J.; Norman, J. E.; Rice, G. E.; Illanes, S. E.	2016	Placenta Journal	DOI: 10.1016/j.placenta.2015.11.015
81	Pathophysiology from preconception, during pregnancy, and beyond.	Hivert, M. F.; Backman, H.; Benhalima, K.; Catalano, P.; Desoye, G.; Immanuel, J.; McKinlay, C. J. D.; Meek, C. L.; Nolan, C. J.; Ram, U	2024	Lancet	DOI: 10.1016/s0140-6736(24)00827-4
82	Vascular endothelial growth factor and its receptors regulation in gestational diabetes mellitus and eclampsia	Bolatai, A.; He, Y.; Wu, N.	2022	Journal of translational medicine	DOI: 10.1186/s12967-022-03603-4
83	Maternal Metabolites Associated With Gestational Diabetes Mellitus and a Postpartum Disorder of Glucose Metabolism.	Liu, Y.; Kuang, A.; Bain, J. R.; Muehlbauer, M. J.; Ilkayeva, O. R.; Lowe, L. P.; Metzger, B. E.; Newgard, C. B.; Scholtens, D. M.; Lowe, W. L.	2021	The Journal of Clinical Endocrinology & Metabolism	DOI: 10.1210/clinem/dgab513
84	Role of Insulin in Placental Transport of Nutrients in Gestational Diabetes Mellitus.	Ruiz-Palacios, M.; Ruiz-Alcaraz, A. J.; Sanchez-Campillo, M.; Larqué, E.	2017	Annals of nutritiont & metabolism	DOI: 10.1159/000455904

85	Fetal programming and gestational diabetes mellitus. *Placenta*	Monteiro, L. J.; Norman, J. E.; Rice, G. E.; Illanes, S. E.	2016	Placenta Journal	DOI: 10.1016/j.placenta.2015.11.015
86	Pathophysiology from preconception, during pregnancy, and beyond.	Hivert, M. F.; Backman, H.; Benhalima, K.; Catalano, P.; Desoye, G.; Immanuel, J.; McKinlay, C. J. D.; Meek, C. L.; Nolan, C. J.; Ram, U.; et al.	2024	Lancet	DOI: 10.1016/s0140-6736(24)00827-4
